#  Causes and Factors Associated with Neonatal Seizure and its Short-term Outcome: A Retrospective Prognostic Cohort Study 

**Published:** 2018

**Authors:** Hamid NEMATI, Parvaneh KARIMZADEH, Minoo FALLAHI

**Affiliations:** 1 Shiraz Neuroscience Research Center, Shiraz University of Medical Sciences, Shiraz, Iran.; 2 Department of Pediatric Neurology, Mofid Children’s Hospital, Faculty of Medicine, Shahid Beheshti University of Medical Sciences, Tehran, Iran.; 3 Pediatric of Neurology Research Center, Shahid Beheshti University of Medical Sciences, Tehran, Iran.; 4 Neonatal Health Research Center, Research Institute for Children Health, Shahid Beheshti University of Medical Sciences, Tehran, Iran.

**Keywords:** Newborn, Seizure, Etiology, Neurodevelopmental outcome

## Abstract

**Objective:**

Neonatal seizures are common, difficult to diagnose and treat, and associated with a great mortality rate and long-term risk of neurodevelopmental impairments. We aimed to determine the etiology, clinical presentation, and neurodevelopmental outcome of neonatal seizures.

**Materials and Methods:**

In this cross-sectional study, 88 neonates, aged < 28 days, admitted to Mofid Children’s Hospital, Tehran, Iran, from September 2011 to 2013 with the initial diagnosis of seizure were enrolled by convenient sampling method. Data, including age, gestational age, birth weight, Apgar of the fifth minute, neonatal intensive care unit (NICU) admission, family history, type, cause, and age of seizure, EEG and paraclinic findings, anticonvulsant used for treatment, neurodevelopmental status, and the final outcome, were collected from medical records and the missed cases were completed by phone calls. The frequency and association of variables were analyzed using SPSS software.

**Results:**

Among neonates with seizures, 67% were male, 79.5% were born term, and 72.7% had normal birth weight. The most common type of seizure was multifocal clonic seizures (45.5%). The main diagnosis in neonates with seizures was hypoxic-ischemic encephalopathy (HIE) (23.9%) and hypoglycemia (10.22%). The mortality rate was 11.36% during a mean follow-up period of 21.4±6.4 months. Neurodevelopmental assessments showed that 64% were normal, 27% had global delay, and 9% had motor delay. Positive family history of epilepsy (*P*=0.006), low Apgar score (*P*=0.002) and epilepsy (*P*<0.001) were significantly associated with adverse neurodevelopmental outcome.

**Conclusion:**

Since HIE and hypoglycemia were the most common cause of neonatal seizures, efforts should be made to improve care during delivery and early breastfeeding.

## Introduction

Despite the tremendous development in medical sciences, and improvement of global health in recent decades, neonatal seizure is still a common phenomenon in neonates and is associated with high rate of morbidity and mortality (1). Neonatal seizure can be a benign transient phenomenon with favorable outcome and no recurrence, but it may be associated with neurodevelopmental disorders or be complicated with epilepsy later in the child’s life in about 30% of survive neonates (2).

Iranian studies have declared a prevalence of 2.4%-9% for neonatal seizure with a mortality rate of about 14% (3, 4), although studies in other countries have reported higher mortality rates (about 17%) (5, 6) and another study found no difference in mortality rate between neonates with seizure and neonates admitted to NICU without seizure (7). Thus, it seems necessary to dig out the reasons underlying the neonatal seizure and factors associated with its mortality.

Several risk factors for its occurrence, including preterm birth, maternal diabetes, and fetal distress, associated with the most common causes of neonatal seizure, including hypoxic-ischemic brain injury and hypoglycemia have been reported (8). Other risk factors for neonatal seizure include maternal hypothyroidism diagnosed after birth (9), and 5-min Apgar scores (10). Genetic factors also play a role in its incidence, as studies have identified the responsible mutations in familial neonatal seizures (11). Besides, abnormal electroencephalography (EEG), and cranial ultrasonography findings and presence of underlying diseases, such as congenital heart disease, have been associated with unfavorable neurodevelopmental outcome (12).

Diagnosis is nevertheless not an easy issue, as the most efficient diagnostic tools, such as video-EEG, are not easily performable (5), and available diagnostic tools, such as computed tomography (CT) and magnetic resonance imaging (MRI) have insufficient accuracy for diagnosis of all lesions (13). In addition, although EEG is the most commonly used diagnostic tool, the accuracy of results are observer-based and various systems have been suggested to increase its performance (14, 15); thus, studies have suggested a higher accuracy in using a combination of methods (13). Initial therapy by common medications seems to be ineffective (16), and the rate of neurodevelopmental disorders and mortality are high. 

Assessing the combination of etiological factors, clinical presentation, different diagnostic tools, and treatments, as well as neurodevelopmental outcome can broad the physicians’ view towards neonatal seizure. Therefore, we aimed to investigate the clinical, paraclinical, and demographic details of neonatal seizure and the association with adverse neurological outcome in a referral pediatric center in Iran.

## Materials and Methods

**Study design**

In this descriptive cross-sectional retro and prospective study, neonates referred to Mofid Children’s Hospital, Tehran, Iran from September 2011 to September 2013 were enrolled. The sample size was calculated at 80 cases and for the probability of lost to follow-up cases, 88 participants were enrolled based on convenient sampling method. All neonates, aged < 28 days, admitted to the hospital with initial diagnosis of seizure by pediatric neurologists and Neonatalogists were included and then followed for neurodevelopmental outcome. Any neonate who was not followed up was excluded from the study. 

Ethics Committee of Shahid Beheshti University of Medical Sciences, Tehran, Iran approved the protocol. The ethical considerations of Helsinki’s declaration were met throughout the study. 

Mofid Hospital is located in central Tehran, Iran, and is considered a referral center for pediatric diseases. Data, including sex, gestational age, age at seizure, birth weight (BW), type of delivery, 5-minute Apgar, NICU admission, need for mechanical ventilation, family history of neonatal seizure, the clinical manifestation of seizure, EEG and paraclinic findings, the status of seizure at the time of discharge, cause of seizure, type of anticonvulsant used for its treatment, neuro/physical developmental status, and the final outcome until discharge, were collected from medical records and the missed cases were completed by phone calls. All participants were followed by phone calls and out-patient visit.

HIE was defined according to the American College of Obstetricians and Gynecologists guidelines. BW was categorized to normal: 2500-4000 g, low (LBW): 1500-2499 g, and very low (VLBW) < 1500 g. As a routine care in this hospital, all EEGs are interpreted by expert child neurologist as normal, mildly abnormal, and moderately-to-severely abnormal and CT, and MRIs are reported by an expert radiologist. To identify the cause of seizure paraclinic tests were performed, including CSF results, electrolytes, metabolic screening tests, and fluid cultures that are examined by Mofid Hospital’s laboratory and are confirmed by the pathologist.

**Statistical analysis**

Descriptive analysis was used for presenting the results, including mean ± standard deviation (SD) for quantitative variables and frequency (percentage) for categorical variables. The association of variables were tested by Fisher’s exact test. For the statistical analysis, the statistical software SPSS software, version 16.0 for windows (SPSS Inc., Chicago, IL, USA) was used. *P* values of 0.05 or less were considered statistically significant.

## Results

 Among neonates with seizures, 66% were male. Most neonates with seizure (79.5%) were term, 12% were born at 34^th^-36^th^ gestational week, and 8% were born < 34^th^ gestational week; mean ±SD of gestational age was 36.99±1.33. Regarding birth weight, 72.7% had normal BW, 15.9% were LBW, 5.7% were VLBW, and 2% weighed over 4 kg, and 3% had IUGR; mean ±SD of birth weight was 2.89±0.58 . Regarding type of delivery, 69% were born through cesarean section, 24% were born by normal vaginal delivery (NVD), and 7% were born by NVD with problems. Only 25% had a positive family history of neonatal seizure.

The Apgar of the fifth minute was 7-10 in 72% of neonates, and < 7 in the rest 28%. Among all neonates, 73% had NICU admission and 23% required mechanical ventilation. Regarding seizure type, the most common type was multifocal clonic seizures (45.5%), 20% tonic, 13% focal clonic, 13% subtle, and 9% myoclonic seizure. The time of occurrence of seizure was in the first 24hours of life in 18.2% of neonates.

The results of EEG showed that 76% had normal to mildly abnormal EEG results and 24% moderately to severely abnormal findings. The results of LP showed that 15% had abnormal findings (pleocytosis, high protein and low sugar), and the results of CT/MRI showed that 50% had abnormal findings. Among abnormal findings of imaging 79.6% were in favor of vascular process (ischemia and stroke) and 4.6% structural brain anomaly and 15.8% unclassified findings.

**Figure 1:**
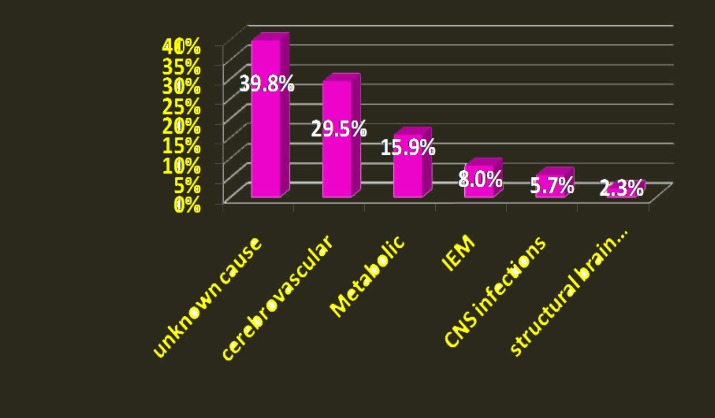
The main etiologies of seizure in the study population: IEM (Inborn Error of Metabolism), Structural Brain Diseases. Vertical( percent).

The main diagnosis in neonates with seizures was cerebrovascular (29.5%) including HIE (23.9%), followed by metabolic (15.9%) including hypoglycemia (10.22%); and inborn error of metabolism (8%): 3 patients with organic academia, 2 with NKH (non-ketotic hyperglycinemia) and 2 with MSUD (maple syrup urine disease). The main etiologies of seizure are demonstrated in Figure 1. As shown, 39.8% were unknown, while the rest of causes were less than 10%. 

**Figure 2:**
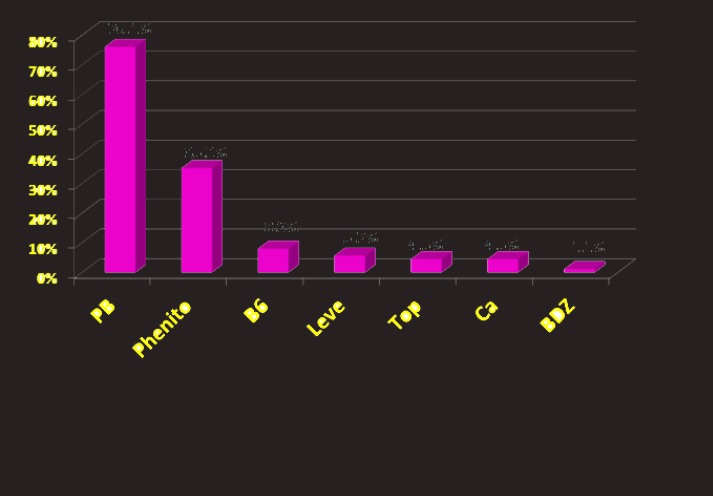
The frequency of drugs used for controlling seizure in the studied patients. PB (Phenobarb), Phenito (Phenytoin), B6 (Vitamin B6), Leve (Levetiracetam), Top (Topiramate), Ca (Calcium), BDZ (Benzodiazepin). Vertical (percent

The main drugs used for controlling seizure included phenobarbital (76.1%), and phenytoin (35.2%), while the rest of drugs had a frequency of less than 10% (Figure 2) and 49% used monotherapy, while 25% used double therapies, and 26% used triple or more therapies. Among all patients, 87% were controlled at discharge and 10.2% were discharged by parents.

**Figure 3:**
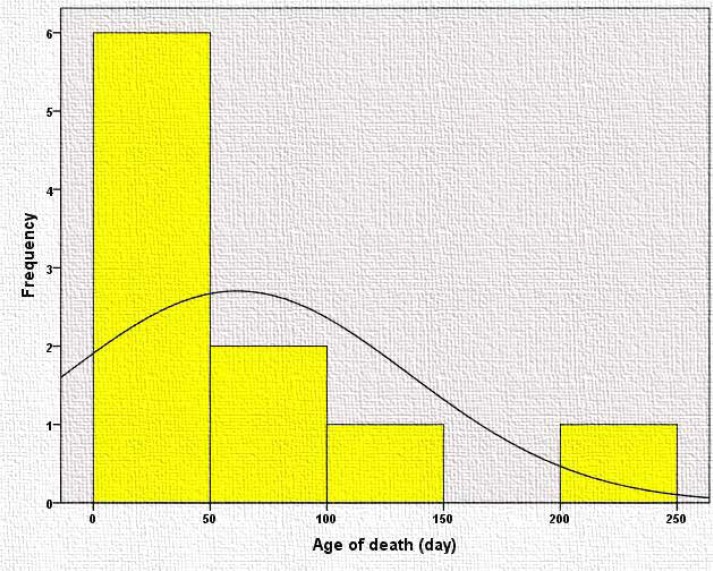
The age of death of patients

**Figure 4:**
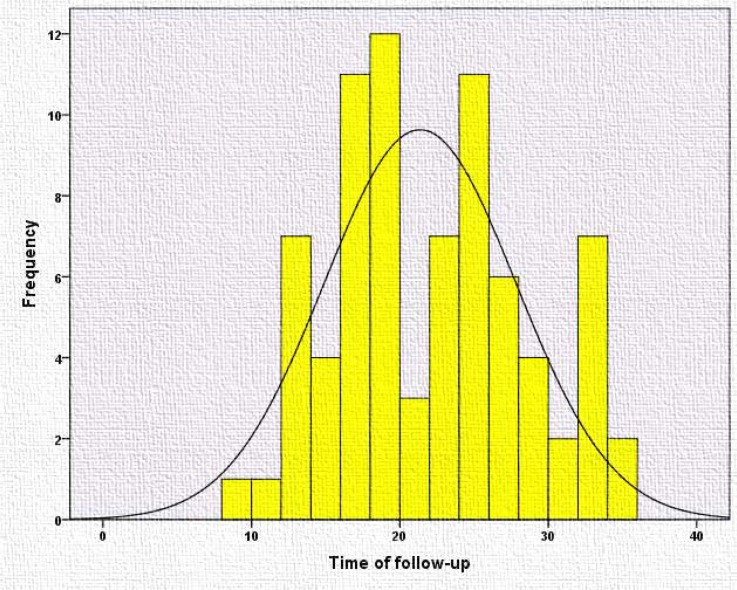
The frequency of duration of follow-up (per month

The mortality rate was 11.36%; the age of neonates at death are demonstrated in Figure 3. Mean follow-up was 21.4±6.4 (range: 9-34) months (Figure 4). During the follow-up period, 23% of neonates had repeated seizures after discharge, while 38% continued their anti-epileptic drug. Neurodevelopmental assessments showed that 64% were normal, 27% had global delay, and 9% had motor delay.

Positive family history of epilepsy, low Apgar score and epilepsy were significantly associated with adverse neurodevelopmental outcome, but the rest of variables were not (Table 1).

## Discussion

The results of the present study indicated a mortality rate of 11.36% during a mean follow-up period of 21.4±6.4 months among 88 neonates with seizures, who were male-dominant, and mostly born term with normal BW. 

The mortality rate reported in the present study is in line with previous Iranian studies, reporting a mortality rate of about 13%-14% (3, 4, 17). It is noteworthy to mention that most studies have established HIE as the most common diagnosis (2-4, 17, 18), which is similar to the results of the present study, indicating the emergent necessity to pay more attention to neonatal/maternal care during delivery in Iran, as HIE is difficult to treat and associated with adverse outcome (19). In addition, the frequency of infection in the present study (5.7%) was lower than the study by Lai et al. in Taiwan (6) and an Iranian study in a rural area (7), both reporting a frequency of nearly 8%, and much lower than the study by Sabzehei and colleagues (24.4%) (3), while the percentage of unknown causes was higher in the present study than previous studies (3, 4, 6), which might be due to the high rate of neonates being discharged by parents (10.2%), and unwillingness to undergo LP (33%), and imaging (15.9%).

**Table 1:** The association of demographic characteristics with neurodevelopmental status

**Variable**	**Category **	**Neurodevelopmental status**		***P*** **-value**
		**Normal, N (%)**	**Delay, N (%)**	
**Sex **	Male	37 (74)	18 (64.3)	0.441
	Female	13 (26)	10 (35.7)	
**Gestational age**	Term	41 (82)	22 (78.6)	0.769
	Preterm	9 (18)	6 (21.4)	
**Type of delivery**	Cesarean section	35 (70)	20 (71.4)	1.00
	NVD	15 (30)	8 (28.6)	
**Apgar of the 5** ^th^ ** minute**	7-10	44 (88)	15 (53.6)	0.002
	<7	6 (12)	13 (46.4)	
**Family history**	Positve	7 (14)	12 (42.9)	0.006
	Negative	43 (86)	16 (57.1)	
**Duration of seizure**	< 24 h	6 (12)	6 (21.4)	0.332
	≥24 h	44 (88)	22 (78.6)	
**Seizure after discharge**	Controlled	47 (94)	13 (46.4)	<0.001
	Repeated	3 (6)	15 (53.6)	

Early-onset seizure was identified as the most common time of occurrence in the present study, which is consistent with the results of previous studies (3, 20). Moreover, male-dominancy of neonates with seizure in the present have been reported in some previous studies (3, 6, 7, 17) that suggests more accurate investigations in male neonates, suspected of seizure. 

The most common type of seizure in the results of the present study was multifocal clonic seizures (45.5%), which is similar to the results of the study by Gebremariam and colleagues, while some studies have reported subtle seizure as the most common type and clonic seizures as the second or third most common type of seizure (3, 4, 6), which might be due to the demographic and epidemiological differences in different studies or the accuracy of different diagnostic tools used. As the type of seizure is associated with long-term neurologic outcome (21), it is suggested that more accurate diagnostic tools be considered for appropriate diagnosis of neonatal seizure.

In the present study, adverse neurodevelopmental outcome occurred in 36% of patients, which was significantly associated with positive family history (*P*=0.006), low 5-minute Apgar score (*P*=0.002), and epilepsy (*P*<0.001) that necessitates proper education for mothers who have positive family history, in addition to paying more attention to neonatal care after delivery. Similarly, previous studies have reported 5-minute Apgar a reliable index of the adverse neurologic outcome (22-24), which is in line with the results of the current study, but another study has confirmed the association between gestational age and adverse outcome (22). Pisani and colleagues have designed a scoring system based on factors associated with adverse neurologic outcome, in order to be able to predict them, which mainly include birth weight, Apgar score, primary neurologic examination, cerebral ultrasound, efficacy of anticonvulsant therapy, and status epilepticus (25). The rate of adverse outcome of neonatal seizure had remained unchanged (18); therefore, it is essential that healthcare staff, including doctors and nurses, pay sufficient attention to the risk factors of neurodevelopmental delay, as proposed in the results of the present study, and educate mothers in this regard, as well, in order to be able to reduce the rate of this morbidity.

As posited, efficacy of anticonvulsant therapy is an important issue in occurrence of adverse neurologic outcome and multiple treatments have been suggested (25, 26), but the results on the most appropriate drug regimen seem to be controversial (27, 28). In the present study, the most commonly used drugs were phenobarbital and phenytoin, which resulted in 36% neurodevelopmental delay, while a higher rate of adverse neurologic outcome are reported by other studies (2, 6, 22). Notably, 13 cases in the present study received no treatment by the neurologist’s advice and stayed without seizure and without neurodevelopmental delay during follow-up. Therefore, it can be concluded that the treatment regimen used by pediatric neurologists in Mofid Hospital seems satisfactory.

Strengths and Limitations: The current study could investigate a combination of clinical, paraclinical, and demographic details of neonatal seizure and the adverse neurologic outcome in neonates referred to a central pediatric hospital. Yet, the present study had some limitations, as well. One of the limitations of the current study was the high rate of neonates being discharged by parents, and unwillingness to undergo further examination that resulted in lack of identification of the cause of seizure in several cases, which might have affected the results regarding the etiology of neonatal seizure. In addition, the retrospective nature of the study, beside the fact that data was collected from one center limited the generalizability of data.

**In conclusion,** the results of the present study indicated HIE and hypoglycemia as the most common causes of neonatal seizures, representing the necessity to take measures to improve care during delivery and early breast-feeding. In addition, the significant association of positive family history and 5-minute Apgar score with neurodevelopmental delay in long-term follow-up demands paying more attention to the maternal/neonatal care during delivery and precise history taking.
